# A disulfide bridge in the calcium binding site of a polyester hydrolase increases its thermal stability and activity against polyethylene terephthalate

**DOI:** 10.1002/2211-5463.12053

**Published:** 2016-04-01

**Authors:** Johannes Then, Ren Wei, Thorsten Oeser, André Gerdts, Juliane Schmidt, Markus Barth, Wolfgang Zimmermann

**Affiliations:** ^1^ Department of Microbiology and Bioprocess TechnologyInstitute of BiochemistryLeipzig UniversityGermany

**Keywords:** biocatalysis, calcium, disulfide bridge, polyethylene terephthalate, protein engineering, protein stability

## Abstract

Elevated reaction temperatures are crucial for the efficient enzymatic degradation of polyethylene terephthalate (PET). A disulfide bridge was introduced to the polyester hydrolase TfCut2 to substitute its calcium binding site. The melting point of the resulting variant increased to 94.7 °C (wild‐type TfCut2: 69.8 °C) and its half‐inactivation temperature to 84.6 °C (TfCut2: 67.3 °C). The variant D204C‐E253C‐D174R obtained by introducing further mutations at vicinal residues showed a temperature optimum between 75 and 80 °C compared to 65 and 70 °C of the wild‐type enzyme. The variant caused a weight loss of PET films of 25.0 ± 0.8% (TfCut2: 0.3 ± 0.1%) at 70 °C after a reaction time of 48 h. The results demonstrate that a highly efficient and calcium‐independent thermostable polyester hydrolase can be obtained by replacing its calcium binding site with a disulfide bridge.

**Abbreviations**
CD: circular dichroism
HEPES: 4‐(2‐hydroxyethyl)‐1‐piperazineethanesulfonic acid
MD: molecular dynamics
PET: polyethylene terephthalate
*p*NPB: *para*‐nitrophenylbutyrate
SD: standard deviation
$T_{\;\;50}^{\;\;60}$: half‐inactivation temperature after 60 min
*T*_*g*_: glass transition temperature
*T*_*m*_: melting point

As a synthetic aromatic polyester with a high melting point (*T*_*m*_), polyethylene terephthalate (PET) has long been considered as recalcitrant to enzymatic degradation 1. A correlation between the *T*_*m*_ of the semi‐crystalline polymer PET and its biodegradability has been postulated 2. However, for a biocatalytic degradation, reaction conditions close to the glass transition temperature (*T*_*g*_) of PET are more relevant 3. At this temperature, the brittle amorphous regions of the polymer become flexible and more accessible to an enzymatic attack 4, 5, 6. Since the *T*_*g*_ of amorphous PET is about 71 °C, the most efficient biocatalytic degradation can be achieved at this temperature or above 4, 7.

Polyester hydrolases from the moderate thermophilic actinomycete *Thermobifida* have been successfully applied for PET surface modification and PET degradation 8, 9, 10. These enzymes, however, cannot be applied at the *T*_*g*_ of PET due to their limited thermal stability. The polyester hydrolase TfCut2 from *Thermobifida fusca* KW3 loses 100% of its activity after 1 h at 65.6 °C 11. A thermal stabilization of the enzyme is therefore required to increase its efficiency for PET degradation.

A calcium‐dependent thermal stabilization of the polyester esterase Est119 from *Thermobifida alba* has been reported previously 12. The analysis of the crystal structure of Est119 revealed a calcium binding site close to its active site 13. Est119 shares a sequence identity of 82% and a highly structural similarity with TfCut2 11. In our previous work, the stabilization effect of calcium in several *T. fusca* polyester hydrolases has been analyzed 14. The *T*_*m*_ of TfCut2 was found to be increased by more than 12 °C in the presence of 10 mm CaCl_2_. Based on molecular dynamics (MD) simulations, the corresponding calcium binding site (D174‐D204‐E253) was modified by substituting its negatively charged amino acid residues with a positively charged arginine. The *T*_*m*_ of the resulting variants D204R and E253R were the same as for the calcium‐stabilized TfCut2. Although PET hydrolysis reactions could be performed by the variants at a reaction temperature of 65 °C in the absence of the dication, the addition of calcium still resulted in higher hydrolysis rates. This finding suggested that calcium is stabilizing the enzyme also at a location not affected by the mutations performed at the calcium binding site 14.

Since the calcium‐induced thermal stabilization of TfCut2 still did not allow the hydrolysis of PET at a reaction temperature close to its *T*_*g*_, we describe here a new approach to engineer its calcium binding site by the introduction of a disulfide bridge in the enzyme structure. A calcium binding site in subtilisin has been previously substituted with a disulfide bridge, resulting, however, in a loss of activity of the enzyme 15. A more recent attempt to replace the effect of calcium with a disulfide bridge in a neutral protease was more successful. However, it involved the fixation of a flexible loop close to the binding site instead of a direct substitution of the binding residues 16.

In this paper, we directly substituted the calcium binding site of TfCut2 with a disulfide bridge to remove its dependence on calcium and show that its thermal stability could thereby be further increased without compromising the hydrolytic activity of the polyester hydrolase.

## Materials and methods

### Materials

Alcalase from *Bacillus licheniformis* was obtained from Sigma‐Aldrich (St. Louis, MO, USA). Low crystalline PET film with a thickness of 250 μm was from Goodfellow Ltd. (Bad Nauheim, Germany, product number 029‐198‐54).

### MD simulations

The disulfide bridge was introduced using the molecular operating environment software package (Chemical Computing Group, Montreal, Canada). The crystal structure of TfCut2 (PDB ID: 4CG1) was used as a template 11. MD simulations were performed using the gromacs 5 software (Uppsala University, Uppsala, Sweden) adopting AMBER99SB force field parameters as described previously 14. Equilibrations and MD simulations were carried out at 373 K. The probability densities of amino acid residues were calculated using GROMACS 5 excluding densities below 5% and those not present in all three rounds of simulation. Occupancy maps were calculated using the vmd 1.9.1 software package (University of Illinois, Champaign, IL, USA) using the same exclusion parameters as applied for the probability densities.

### Site‐directed mutagenesis

Mutations were introduced using the Q5 Site‐Directed Mutagenesis Kit (New England Biolabs, Ipswich, MA, USA). Mutagenesis results were verified by sequencing.

### Enzyme production and purification

The proteins of the genes encoding TfCut2 (GenBank ID: FR727681) and all generated variants were recombinantly produced in *E. coli* BL21(DE3) using the pET‐20b(+) vector (Novagen, Darmstadt, Germany) 11. Recombinant proteins were purified by affinity chromatography in imidazole buffer using Ni‐NTA resin (Qiagen, Hilden, Germany) followed by size exclusion chromatography (Superdex 200; GE, Munich, Germany) with sodium borate buffer (50 mm, pH 8) as an eluent 14. Protein concentrations were estimated by a modified Bradford method 17.

### Determination of half‐inactivation temperatures of the enzymes

To determine half‐inactivation temperatures ($T_{50}^{60}$), the residual esterase activity of the enzymes was determined 18. Purified enzyme solutions (10 μg·ml^−1^) were incubated for 60 min at temperatures between 63 and 87 °C in an ep gradient S thermocycler (Eppendorf, Hamburg, Germany) in sodium borate buffer (50 mm, pH 8) with or without the addition of CaCl_2_ (10 mm). The incubation was stopped by cooling the samples to 4 °C. The residual activity was determined in HEPES buffer (0.1 m, pH 8) with 0.5 mm *p*NPB. The enzymatic hydrolysis of *p*NPB was spectrophotometrically monitored for 5 min at 405 nm in a microplate reader (Biotek Power Wave XS, Winooski, VT, USA). All determinations were performed in triplicates. Half‐inactivation temperatures were calculated by nonlinear regression as described previously 18.

### Circular dichroism spectroscopy

Thermal denaturation experiments were performed on a Jasco J‐715 spectropolarimeter using CD spectroscopy (JASCO, Easton, PA, USA). Protein solutions (3 μm) in sodium borate buffer (50 mm, pH 8) were prepared in quartz cuvettes with a path length of 2 mm (Hellma, Jena, Germany) with or without CaCl_2_ (10 mm). Thermal denaturation was determined by recording the ellipticity at a wavelength of 222 nm. The temperature was increased continuously (5 °C·min^−1^) from 50 to 98 °C. Ellipticity data were recorded in 1 °C steps. These data were normalized to the corresponding values at 50 and 98 °C. *T*_*m*_ values were calculated applying a two‐state nonlinear regression with linear pre‐ and post‐transition changes as described previously 19.

### Enzymatic hydrolysis of PET films

Pieces of PET film (45 mg, 3 cm^2^ surface area) were prepared as described previously 14. Each film was placed in a 1.5 mL reaction tube with screw cap containing 1.5 mL of HEPES buffer (0.5 m, pH 8), purified enzyme (50 μg) and with or without CaCl_2_ (10 mm). Negative controls were prepared similarly with enzyme solution replaced by sodium borate buffer (50 mm, pH 8). Samples were incubated for 48 h on a thermoshaker TS1 (Biometra, Göttingen, Germany) (1000 r.p.m.) at 65, 70, 75, and 80 °C. The reactions were stopped by incubating the films in phosphate buffer (100 mm, pH 8) containing 0.1 U *B. licheniformis* alcalase for 15 min at 50 °C. Weight losses of the films were determined gravimetrically before and after incubation with the enzymes as previously described 14. All determinations were performed in triplicates.

### Determination of temperature optimum of the enzymes

For the determination of the optimum reaction temperature for PET hydrolysis, pieces of PET film (15 mg, 1 cm^2^ surface area) were washed and dried as described previously 14. Each film was placed in a 0.3 mL reaction tube containing 230 μL of HEPES buffer (0.5 m, pH 8 with NaOH), purified enzyme (16.7 μg), and CaCl_2_ (10 mm). Negative controls were prepared similarly with enzyme solution replaced by sodium borate buffer (50 mm, pH 8). Samples were incubated for 60 min on a thermoshaker TS1 (Biometra) (1400 r.p.m.) at 60, 65, 70, 75, 80, and 85 °C. The reactions were stopped by cooling to 4 °C. Samples were prepared and the formation of hydrolysis products was monitored by reversed phase high performance liquid chromatography as described elsewhere 20. All determinations were performed in triplicates.

### Statistics and data analysis

Significance levels were calculated using the Tukey method as part of the graph pad prism 5 software package (Graph Pad Software, La Jolla, CA, USA). Nonlinear regression analysis was performed using sigma plot 11 software package (Systat, San Jose, CA, USA).

## Results

### Half‐inactivation temperature and melting points of the TfCut2 disulfide bridge variants

**Figure 1:**
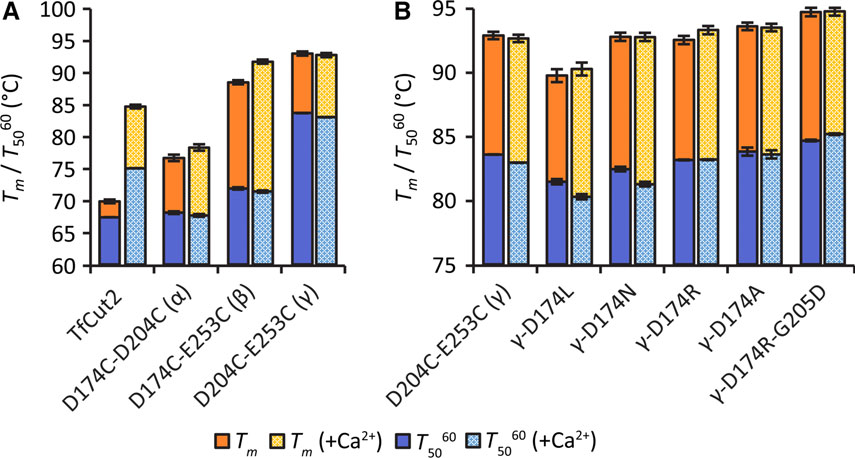
Melting points (*T*_*m*_) and half‐inactivation temperatures ($T_{50}^{60}$) of TfCut2 and its calcium binding site variants. The *T*_*m*_ was determined by CD spectroscopy at a wavelength of 222 nm in sodium borate buffer (50 mm, pH 8) with and without 10 mm CaCl_2_. The $T_{50}^{60}$ was determined by residual activity measurements against *p*NPB (0.5 mm, pH 8) in sodium borate buffer (50 mm, pH 8) with and without 10 mm CaCl_2_. All values were calculated by nonlinear two‐state regressions performed in triplicate. Error bars represent standard deviations of the triplicate determinations. Values are grouped in disulfide bridges of the original calcium binding site (A) and mutations of the disulfide bridge variant D204C‐E253C (γ) (B).

To remove the calcium dependence of TfCut2 and its variants containing a salt bridge in the calcium binding site for their thermal stability, the effect of introducing a second disulfide bridge was analyzed. To determine an optimum replacement of the calcium binding site, all combinations of the three binding residues D174, D204, and E253 were substituted by a disulfide bridge. Analysis of the wild‐type TfCut2 and the variants obtained showed a *T*_*m*_ of 69.8 ± 0.3 °C (SD) and a $T_{50}^{60}$ of 67.3 ± 0.0 °C (SD) without and a *T*_*m*_ of 84.6 ± 0.1 °C and a $T_{50}^{60}$ of 74.9 ± 0.1 °C in the presence of 10 mm CaCl_2_ for the wild‐type enzyme (Fig. 1A). Similar *T*_*m*_ and $T_{50}^{60}$ values were obtained with the variant D174C‐D204C (α) with or without the addition of calcium. While the $T_{50}^{60}$ values showed no difference from the wild‐type TfCut2, the *T*_*m*_ value without calcium was 9.7% higher, however, still lower compared to the calcium‐dependent wild‐type enzyme. The variant D174C‐D253C (β) showed a strongly increased *T*_*m*_ of 88.3 ± 0.3 °C without and 91.6 ± 0.6 °C in the presence of calcium. However, only a moderate $T_{50}^{60}$ increase of 6% compared to the wild‐type enzyme was observed. A disulfide bridge at D204C‐E253C (γ) resulted in a significant increase of both the *T*_*m*_ and the $T_{50}^{60}$ of 92.8 ± 0.3 °C and 83.5 ± 0.0 °C, respectively. A thermal stabilization of the enzyme that was completely independent from calcium was obtained with this variant.

### MD simulations

**Figure 2:**
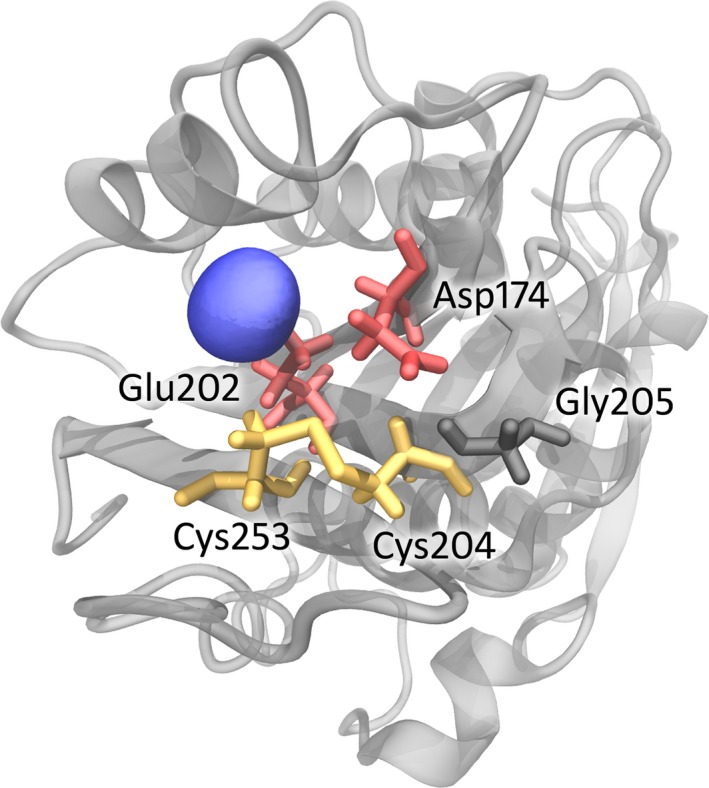
Binding of Ca^2+^ to the TfCut2 variant D204C‐E253C (γ) during a 50 ns MD simulation. The distribution of Ca^2+^ is represented as blue spheres and was calculated as averaged occupancy maps within a radius of 3.7 Å obtained from triplicate simulations. The occupancy map is limited to a minimum level of 5% obtained in all simulations. The protein backbone is highlighted in gray, the disulfide bridge D204C‐E253C in yellow, binding residues close to the original binding site in red and nonbinding residues of the original binding site in black.

MD simulations were performed to analyze the binding of calcium by the TfCut2 variants containing a modified binding site at a high temperature (100 °C). Three independent simulations of 50 ns were carried out with the variant D204C‐E253C in the presence of 10 mm CaCl_2_. When the probability density of Ca^2+^ within a radius of 3.7 Å to D204C‐E253C was calculated, E26 with a probability density of 42.5 ± 21.0% (SD), D174 with 27.8 ± 20.0%, E202 with 22.4 ± 18.4% and E64 with 21.4 ± 10.4% were observed in close vicinity to the dication. The occupancy map of Ca^2+^ in D204C‐E253C revealed that the binding occurred close to the original calcium binding site D174‐D204‐E253 14 (Fig. 2). At this position, Ca^2+^ was bound to both D174 and E202.

Since Ca^2+^ was found still to be bound to D174 at the original binding site, simulations were performed with additional mutations of D204C‐E253C. D174A was selected to reduce the steric hindrance, D174L to substitute with a lipophilic residue of similar size, D174N to remove charge by retaining the size, D174R to change to a positive charge and D174R‐G205D to introduce an additional salt bridge imitating the adjacency of the vicinal loops caused by calcium. None of these variants showed a Ca^2+^ ion bound close to the binding site at D174 or E202. Instead, binding occurred at E64 and D85 with D174A, at D246 with D174L, at E64, D85 and E26 with D174N, E26 and E72 with D174R and E26, P263 and E72 with D174R‐G205D. The prevalence of E26 and E72 as binding residues was probably caused by their vicinal position in the protein. However, these residues were located at a distance that did not allow for the introduction of a disulfide bridge. E64 and D85 were located close enough to each other to introduce a disulfide bridge. However, D85 may form a salt bridge with R31 and was therefore not considered for a replacement.

### Half‐inactivation temperature and melting points of the D204C‐E253C variants

Based on the results obtained by the MD simulation experiments, D204C‐E253C (γ) was selected for further mutations. To eliminate potentially destabilizing effects at the remaining D174 residue of the original calcium binding site, further mutations were introduced at this position. The elimination of D174 as a steric hindrance by γ‐D174A or the substitution with a positively charged residue to obtain γ‐D174R revealed no significant differences in the *T*_*m*_ and $T_{50}^{60}$ values compared to D204C‐E253C (γ) (Fig. 1B). Similarly, a reduction in the negative charge by introducing small structural changes to obtain γ‐D174N showed no effect on the *T*_*m*_ while the $T_{50}^{60}$ even decreased significantly (*P* < 0.001). Furthermore, a substitution by the nonpolar γ‐D174L resulted in a significant decrease of *T*_*m*_ and $T_{50}^{60}$ (*P* < 0.001). In contrast, the introduction of a salt bridge between D174R and G205D to mimic the attachment of the loops caused by calcium was more successful. With a significantly increased *T*_*m*_ of 94.6 ± 0.6 °C (*P* < 0.01) and $T_{50}^{60}$ of 84.6 ± 0.5 °C (*P* < 0.001) without calcium, γ‐D174R‐G205D represented the TfCut2 variant obtained with the highest thermal stability and concomitant calcium independence.

### Hydrolysis of PET films by the TfCut2 disulfide bridge variants

**Figure 3:**
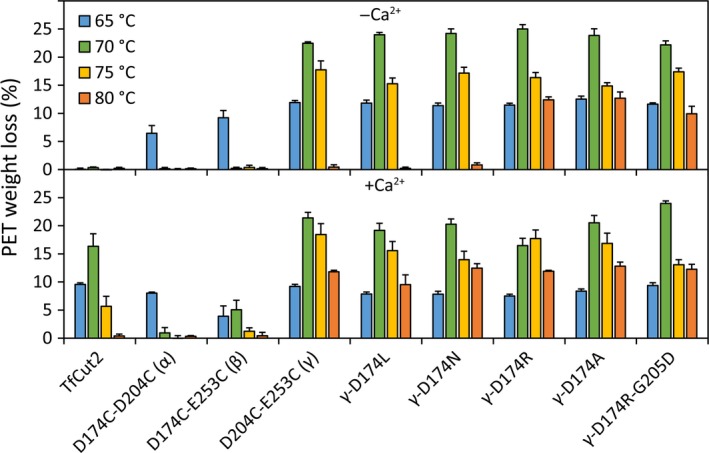
Weight loss of PET films hydrolyzed by TfCut2 variants at 65, 70, 75, and 80 °C for 48 h. The reactions were carried out in HEPES buffer (0.5 m, pH 8) with and without CaCl_2_ (10 mm). Error bars represent standard deviations of triplicate determinations.

While the wild‐type TfCut2 caused no considerable weight loss of PET films during a reaction time of 48 h at temperatures between 65 and 80 °C, the addition of 10 mm CaCl_2_ resulted in weight losses at reaction temperatures up to 75 °C with a maximum of 16.3 ± 2.2% (SD) obtained at 70 °C (Fig. 3). The variants D174C‐D204C (α) and D174C‐E253C (β) caused weight losses of 6.5 ± 1.4% and 9.2 ± 1.3% at 65 °C without, as well as 8.0 ± 0.2% and 3.9 ± 1.8% in the presence of 10 mm calcium. D204C‐E253C (γ) caused the highest weight loss among variants carrying solely a disulfide bridge 22.5 ± 1.6% without and 21.4 ± 1.0% with 10 mm calcium at 70 °C.

Among the D204C‐E253C (γ) derivatives, γ‐D174L, γ‐D174A and γ‐D174R‐G205D showed no significantly different weight loss at 70 °C without calcium compared to their template protein D204C‐E253C. In contrast, the charge‐reduced γ‐D174N and the positively charged γ‐D174R exhibited a higher weight loss of 24.2 ± 0.8% (*P* < 0.05) and 25.0 ± 0.8% (*P* < 0.001) at the same conditions. While the weight loss in the presence of calcium decreased by 3.3‐8.5% with γ‐D174A, γ‐D174L, γ‐D174N and γ‐D174R, it increased moderately by 1.8% with γ‐D174R‐G205D.

At 75 °C, the overall weight loss (13.0–18.4%) of the γ‐derivatives decreased compared to 70 °C. The presence of calcium reduced the weight loss with γ‐D174N and γ‐D174R‐G205D by 3.2–4.3%, improved it moderately with γ‐D174A and γ‐D174R by 1.4–2.0% and showed no change with γ‐D174L. At 80 °C, D204C‐E253C (γ), γ‐D174L, and γ‐D174N only caused a weight loss in the presence of calcium (9.5–12.5%). In contrast, the variants γ‐D174R, γ‐D174A, and γ‐D174R‐G205D caused a considerable weight loss of PET films in the presence (11.9–12.8%) as well as in the absence (9.9–12.7%) of calcium.

### Temperature optimum of the TfCut2 disulfide bridge variants for the hydrolysis of PET films

**Figure 4:**
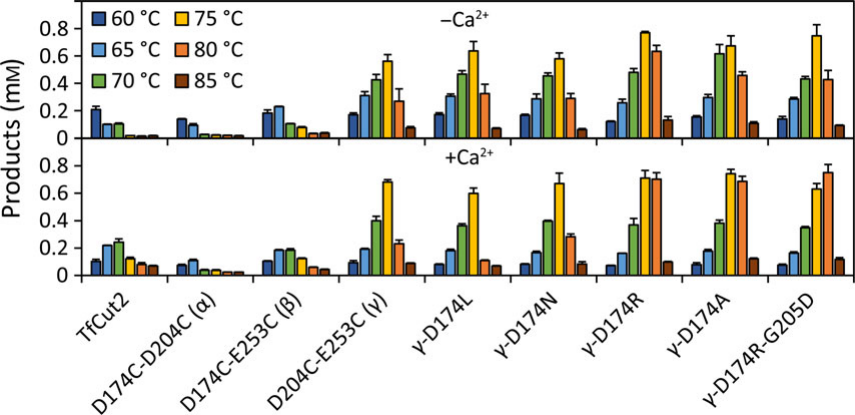
Optimum temperature of the TfCut2 variants for PET hydrolysis. The reactions were carried out at temperatures from 60 to 85 °C for 60 min in HEPES buffer (0.5 m, pH 8) with and without CaCl_2_ (10 mm). Error bars represent standard deviations of triplicate determinations.

As indicated by the gain of the *T*_*m*_ and the $T_{50}^{60}$ values, the thermal stability could be considerably increased by mutations of the calcium binding site. The reaction temperature optimum of the variants for the hydrolysis of PET films was analyzed by determining the total amount of water‐soluble hydrolysis products released during a reaction time of 60 min (Fig. 4). The wild‐type TfCut2 showed the highest product formation (0.21 ± 0.02 mm) without calcium at 60 °C. The addition of calcium resulted in a shift of the optimum reaction temperature to 70 °C and a product formation of 0.24 ± 0.02 mm. The variants D174C‐D204C (α) and D174C‐E253C (β) showed a maximum PET hydrolysis activity between 60 and 65 °C. A calcium‐induced shift of the optimum reaction temperature to 70 °C was only observed with D174C‐E253C (β). The variant D204C‐E253C (γ) exhibited an optimum reaction temperature at 75 °C and also showed a strongly increased product formation of 0.56 ± 0.05 mm and 0.68 ± 0.02 mm without and in the presence of calcium, respectively.

The derivatives of D204C‐E253C (γ) showed a similar optimum reaction temperature at 75 °C without calcium except for γ‐D174A, which displayed an optimum temperature range between 70 and 75 °C. In the presence of calcium, the optimum reaction temperature of the variants γ‐D174R, γ‐D174A, and γ‐D174R‐G205D was even further shifted to 75–80 °C. The maximum product formation in the presence of calcium was detected at 80 °C with γ‐D174R‐G205D (0.75 ± 0.06 mm) and at 75 °C with γ‐D174A (0.74 ± 0.03 mm). In the absence of calcium, the highest product formation (0.77 ± 0.01 mm) was determined with γ‐D174R at 75 °C.

## Discussion

By the replacement of the calcium binding site by a disulfide bridge, the calcium dependence of the polyester hydrolase TfCut2 from *T. fusca* KW3 could be removed while retaining its thermal stability and hydrolytic activity against PET. The *T*_*m*_ and the $T_{50}^{60}$ values of the disulfide bridge variants were indeed increased by up to 24.9 and 17.3 °C, respectively. Compared to a previously reported increase of *T*_*m*_ by 14 °C effected by a substitution of the calcium binding site with a salt bridge, a further gain of more than 10 °C in the *T*_*m*_ could thus be obtained 14. The most stable variant has the D204C‐E253C disulfide bridge. Likewise, the introduction of a salt bridge at the same positions also resulted in the highest increase in thermal stability 14.

The H208 residue located on the same loop as D204 has been observed to move outwards from the active site when the temperature is increased in MD simulations with a homologous polyester hydrolase 21. With the variants D174C‐D204C (α) and D174C‐E253C (β), we could expand the postulated role of the active site loop with the catalytic H208 in the thermal inactivation process of the enzyme.

The variant D174C‐E253C showed a considerable gain of its *T*_*m*_, however, only a small increase in its $T_{50}^{60}$ value. While the overall structure of the protein was stabilized by the introduction of the D174C‐E253C disulfide bridge, the active site was still prone to a thermal inactivation due to the flexible H208 loop. Interestingly, D174C‐D204C (α) showed no change in $T_{50}^{60}$ and only a small increase in *T*_*m*_. A connection from D204 to E253 is obviously essential for the overall structural integrity of the protein at higher temperatures. A stabilization of the H208 loop is therefore likely to show an effect only when the whole protein is structurally further secured.

The variant D204C‐E253C (γ) carried the most effective substitutions for the two calcium binding residues of the wild‐type enzyme. When potential interactions between the remaining binding residue D174 and the disulfide bridge were analyzed, the mutation of the position D174 in the variant D204C‐E253C (γ) resulted in only moderate effects on the thermal stability and enzyme activity. Only by the introduction of a salt bridge between D174R and G205D a small increase in *T*_*m*_ and $T_{50}^{60}$ by 1–2 °C was observed.

Although the effects of mutations at the residue D174 on the stability of the protein was only moderate, these modifications considerably influenced the hydrolytic activity of the variants against PET. While the weight losses of PET films hydrolyzed by γ‐D174R were increased by up to 10% compared to D204C‐E253C (γ) at 70 °C, a 28‐fold increase was detected at 80 °C. At this temperature, γ‐D174A caused a 28‐fold, and γ‐D174R‐G205D a 22‐fold higher weight loss of PET films compared to D204C‐E253C (γ).

The increased PET hydrolytic activity of the variants γ‐D174R and γ‐D174R‐G205D could be a result of a reduced degree of freedom of D174R due to its interaction with the negatively charged E202 or G205D. The remaining calcium binding residue D174 may therefore represent a steric hindrance promoted by its negative charge that is not stabilized by vicinal positively charged residues. However, the Ca^2+^ dependance of γ‐D174N at 80 °C indicates that the charge of D174 exerts a smaller influence than its sterical hindrance. Since the *T*_*m*_ and $T_{50}^{60}$ values of the D204C‐E253C (γ) variant did not indicate a further stabilization effect, the higher PET hydrolysis activity of γ‐D174R and γ‐D174A compared to this variant cannot be explained by a denaturation or inactivation of the enzyme.

A comparison of the optimum temperature for PET hydrolysis by the variants indicated a possible reason for their detected higher activity. The temperature optima shifted to higher temperatures when D204C‐E253C (γ) was mutated to γ‐D174A, γ‐D174R, or γ‐D174R‐G205D. These results indicate an interference between D174 and the disulfide bridge which intensified at higher temperatures. Since mutations resulting in a reduced steric hindrance were found to be advantageous for a hydrolytic activity at 80 °C, a mutation of the third binding residue D174 would be required to avert its negative effect on the activity of the enzyme. The variant γ‐D174R showed the highest hydrolytic activity against PET films and represented the most efficient structure for the replacement of the calcium binding site of TfCut2 with a disulfide bridge.

An optimum reaction temperature between 75 and 80 °C could be achieved with the variants γ‐D174R, γ‐D174R‐G205D, or γ‐D174A. This increase in thermal stability can probably not be extended by a further redesign of the calcium binding site. By the introduction of a disulfide bridge, it has been possible to raise the thermal stability of TfCut2 to enable the hydrolysis reaction to be performed at a favorable temperature close to the *T*_*g*_ of PET 4. Furthermore, the dependence of the enzyme on calcium could be completely removed. The strategy applied for the polyester hydrolase TfCut2 provides an efficient approach to stabilize proteins with an analogous structure of their calcium binding site to increase the thermal stability without negatively influencing the activity of the enzyme.

## Author contributions

JT and WZ were responsible for study conception and design. RW and TO revised the manuscript. AG assisted mutagenesis experiments. JS assisted protein expression and MB assisted HPLC experiments.
